# Clinical Features and Treatment in the Spectrum of Paroxysmal Dyskinesias: An Observational Study in South-West Castilla y Leon, Spain

**DOI:** 10.1155/2019/4191796

**Published:** 2019-05-02

**Authors:** Raquel Manso-Calderón

**Affiliations:** ^1^Department of Neurology, University Hospital of Salamanca, 37007 Salamanca, Spain; ^2^Instituto de Investigación Biomédica de Salamanca (IBSAL), 37007 Salamanca, Spain

## Abstract

**Background:**

Paroxysmal dyskinesias (PxD) are a group of heterogeneous disorders characterized by intermittent episodes of involuntary movements. PxD include paroxysmal kinesigenic (PKD), nonkinesigenic (PNK), and exercise-induced (PED) varieties.

**Objectives:**

To define the phenotype of primary and secondary PxD forms.

**Methods:**

Twenty-two patients with PxD (9 men/13 women) were evaluated in two hospitals in south-west Castilla y Leon, Spain. Clinical features of the episodes, causes, family history, and response to treatment were collected.

**Results:**

Thirteen participants with primary PxD (6 men/7 women) and 9 with secondary PxD (3 men/6 women) were recruited. Nine patients belong to three nonrelated families (2 had PKD and 1 had PED). Mean age at onset in primary PKD cases was 10 years (range 5-23 years), earlier than in PNKD (24 years) and PED (20 years). Most primary PKD cases experienced daily episodes of duration <1 minute, which are more frequent and shorter attacks than in PNKD (1-2 per month, 5 minutes) and PED (1 per day, 15 minutes). The location of the involuntary movements varied widely; isolated dystonia was more common than mixed chorea and dystonia. All PKD patients who received antiepileptic treatment significantly improved. Levodopa and ketogenic diet proved to be effective in two patients with PED. Secondary forms presented a later mean age of onset (51 years). Six cases had PNKD, 1 had PKD, 1 both PNKD and PKD, and 1 had PED. Causes comprised vascular lesions, encephalitis, multiple sclerosis, peripheral trauma, endocrinopathies, and drugs such as selective serotonin reuptake inhibitors (SSRIs).

**Conclusion:**

The knowledge of the clinical features and spectrum of causes related to PxD is crucial to avoid delays in diagnosis and treatment, or even a nonorganic disorder diagnosis.

## 1. Introduction

Paroxysmal dyskinesias (PxD) comprise a group of heterogeneous syndromes characterized by recurrent attacks of involuntary movements—typically dystonia, chorea or a combination of them—without loss of consciousness [1]. However, “paroxysmal dyskinesias” constitute an ambiguous definition, because the term “paroxysmal” etymologically refers to sudden attacks, recurrence, or intensification of a disease, whereas “dyskinesias” have different meanings, including an impairment of the ability to execute voluntary movements or involuntary jerky movements with a fixed pattern. In this sense, conditions such as tic-syndromes, action-myoclonus, or action-tremor, that do not match with the pretended meaning that movement disorders experts give to PxD, would fit into this category [2].

Furthermore, the PxD classifications have changed over the years. Firstly, the episodes were catalogued depending on the duration (short, < 5 minutes; long, > 5 minutes) [3]. Following this former classification, Dermikian and Jankovic proposed three different subtypes based on triggers: paroxysmal kinesigenic (PKD), nonkinesigenic (PNKD), and exercise-induced (PED) dyskinesias [4]. A fourth subtype, hypnogenic paroxysmal dyskinesia (HPD), is thought to be a form of nocturnal frontal lobe epilepsy [5]. Noteworthy, in recent years, the differentiation of subgroups depending on the etiology (primary and secondary disorders) has gained relevance. Primary disorders can include those cases where no definite causes for PxD have been found, labeled as idiopathic forms, and those with a specific genetic mutation established (i.e., PRRT2-PKD or SLC2A1-PED) [2, 6]. In some cases a specific cause for the PxD has been identified, such as multiple sclerosis, vascular lesions, trauma, or metabolic disorders [7].

The prevalence of PxD is not clearly defined, but some authors have reported a prevalence lesser than 1% [7]. However, PxD are probably underdiagnosed because it is common that nobody witnesses the episodes of PxD due to its short duration. In addition, the common lack of abnormal signs between the attacks, especially in primary forms, increases the diagnosis challenge [1]. Therefore, recognition of characteristic descriptions, encompassing triggers and clinical features of the attacks, could lead us to conduct the appropriate investigations in order to reach a definite diagnosis, on which treatment is highly dependent [2].

## 2. Subjects and Methods

Twenty-two patients diagnosed with PxD were recruited from the Hospital Nuestra Señora de Sonsoles (Avila) between 2009 and 2011 and from the University Hospital of Salamanca (Salamanca) between 2012 and 2016, both in the region of Castilla y Leon, Spain. Inclusion criteria were (1) evidence of PxD by examination and/or video review (i.e., recorded using a mobile phone), with or without previous medical history; and (2) evidence of abnormal involuntary movement with an episodic nature, sudden onset, and not associated with a change in consciousness or seizure activity [4, 7].

Psychogenic/functional causes were excluded based on the existence of psychiatric disorders, profound within-subject phenomenological or attack duration variability, description of several and nonspecific triggers, frequent alteration of responsiveness during attacks, medically unexplained somatic or neurological symptoms, and atypical response to medications, including, in some cases, improvement of symptoms with placebo [8–10].

All patients were evaluated by a neurologist (RMC) and classified according to the previous reported classification as follows: PKD was diagnosed if the involuntary movements were triggered by an abrupt voluntary movement, PNKD was diagnosed if there was no clear triggering factor for the episodes (i.e., the involuntary movements occurred spontaneously at rest), and PED was assumed when the attacks were preceded by prolonged physical exertion [4].

The following data were collected: diagnosis, sex, age at onset, type of movement, duration and frequency of symptoms, presence of auras or pain, location of movement, precipitants, exacerbating factors, proven treatments, and response. In addition, PxD were categorized by etiology as (1) primary—familial or sporadic—or (2) secondary. For a familial origin, a positive family history of dyskinesias was required. Brain computerised tomography (CT) and/or magnetic resonance imaging (MRI) as well as electroencephalography (EEG) was obtained of the index cases of each family and all the sporadic and secondary cases, except for a participant with encephalitis due to systemic candidiasis, who died before completing the study. Some of them were also checked with a brain single photon emission computed tomography (SPECT). The results were compared with findings reported in the literature.

## 3. Results

### 3.1. Primary Paroxysmal Dyskinesias

The present study consists of 13 patients with diagnosis of primary PxD (6 men/7 women) [Table 1]. Nine participants belong to three unrelated families (two families had PKD [F1 and F2] and one had PED [F3]) and four cases are sporadic (one had PKD [E1], two had PNKD [E2 and E3], and one had PED [E4]). The mean age at onset of the participants with primary PKD was 10 years (range 5-23 years), much earlier than in those cases categorized as PNKD with 24 years (range 16-33 years). In the PED group, the age at onset differed significantly between the familial cases (24 years) and the sporadic one (7 years). Most subjects categorized as primary PKD experienced daily attacks—at the time with the largest number of episodes—with duration of less than 1 minute, while those with PNKD reported one or two episodes per month that last 5 minutes and individuals with PED suffered an average of one attack in a day lasting 15 minutes. The location of involuntary movements varied widely, and pure dystonia predominated over mixed chorea and dystonia. In all PKD patients who received antiepileptic treatment was observed a significantly decrease in the number of attacks. A familial case affected by PED achieved a favourable response to levodopa, whereas the patient with sporadic PED improved after starting a ketogenic diet.

**Figure 1:**
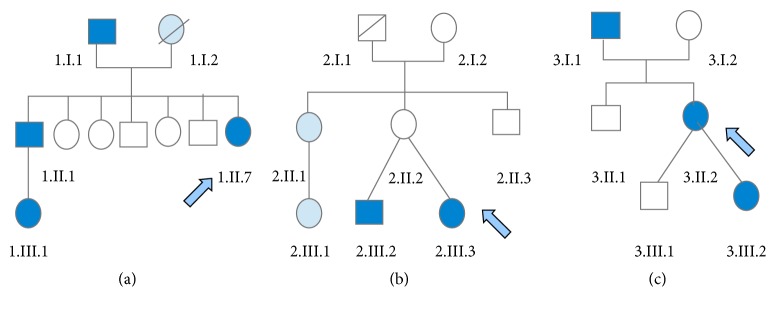
Family trees of the three studied families: families 1 (a), 2 (b), and 3 (c). The members affected by PxD are displayed shaded in dark blue and those affected by epilepsy, in light blue. The cases indicated with an arrow correspond to the index cases.

Neuroimaging, electromyography, and electroencephalography (EEG) studies were normal in all cases, with the exception of an EEG with sleep deprivation carried out in a patient with PNKD [E2], in which generalized paroxysmal discharges of sharp waves with large amplitude and short duration were observed, but that were not accompanied by objective clinical manifestations. Two PKD cases [F1. II.7 and E1] associated migraine with aura; one with PKD [F1. II.7] presented a restless legs syndrome; and another with PED [F3. II.2] reported both migraine without aura and cramps with exercise. The two families with PKD [F1 and F2] had a history of epilepsy [see Figure 1]. One of the families with PKD [F1] was of Uruguayan ancestry, and one of the cases with PNKD [E2], of Moroccan origin. All cases, but the last one [E4], were diagnosed for the first time of PxD in our consultation.

### 3.2. Secondary Paroxysmal Dyskinesias

The study includes 9 cases with secondary PxD (3 men/6 women) [Table 2], with a mean age at onset much later (51 years) than in the primary ones. Six cases had PNKD, one had PKD, one had mixed symptoms, and another one had PED. Causes include vascular lesions (significant cerebrovascular disease (CVD) [S2] and right parietal haemorrhage [S8]), demyelinating diseases (relapsing-remitting multiple sclerosis (MS) with 9 years of evolution and residual paresis and right brachial hypoesthesia [S1]), infectious (haemorrhagic leukoencephalitis due to toxoplasmosis in an HIV patient without pretreatment [S3] and encephalitis due to systemic candidiasis [S6]), peripheral trauma (right ankle dislocation) [S9], endocrine disorders (hypoparathyroidism) [S7], and drugs (fluoxetine [S4] and escitalopram [S5]).

The majority of the cases presented movements of unilateral location; only a case with PKD/PNKD experienced a progression from unilateral to bilateral distribution.

**Figure 2:**
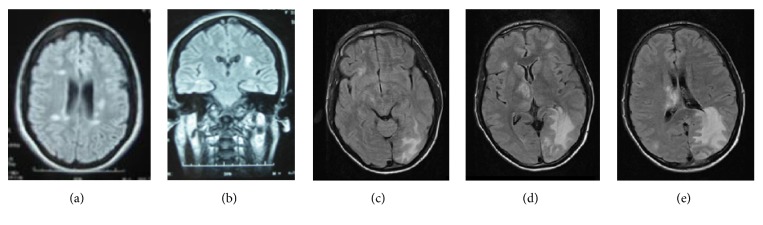
Brain MRI showed demyelinating lesions in a patient with multiple sclerosis (a, b) [S1] and haemorrhagic leukoencephalopathy due to toxoplasmosis in HIV patient (c, d, e) [S3].

The frequency and duration of the attacks were variable, as well as the response to treatment. EEG studies evidenced no epileptiform activity. Neuroimaging studies showed multiple demyelinating lesions in frontal and parietal bilaterally and in brainstem in the patient with MS [S1]; ischemic periventricular leukoencephalopathy and lacunar infarctions in bilateral basal ganglia in that one with CVD [S2]; haemorrhagic leukoencephalitis with two haemorrhagic lesions in the right thalamic area in the case with HIV [S3]; and a right parietal haematoma in the patient with the cerebral haemorrhage [S8], being normal in the rest [see Figure 2]. The patient with encephalitis due to systemic candidiasis [S6] died before completing the study.

## 4. Discussion

In the cases with familial primary PxD, the delay in diagnosis is remarkable since the onset of the clinic, favoured by the intermittent nature of the episodes. In fact, only in a case with primary PxD an attack was witnessed “*in situ*” (two episodes of PKD at the entrance to the consultation and after walking to the examination table for lying down, with a refractory period of 20 minutes in between) without exert intentional provoked manoeuvres. In order to characterize the type of dyskinesia (dystonia, chorea, etc.) can be useful ask the patient that imitates the movements of the (s) limb (s) affected (s) during the attacks or that someone records them using video (e.g., by means of a mobile device). The classification of a case as having PKD or PED sometimes is difficult, especially when the clinical features are limited to upper extremities, resulting essential in the differential diagnosis the duration of both the trigger effort and the attacks [11]. In terms of overlap with other syndromes, in this series a family [F2] with infantile convulsions and paroxysmal choreoathetosis (ICCA) was found, an association commonly observed in PRRT2 mutations [5, 6, 12]. The case suffering from epilepsy in the F1 family was of vascular origin, so this family was considered to present a pure form of PKD.

In the cases with PKD Bruno's diagnostic criteria were met: kinesigenic trigger, duration < 1 min in the majority of attacks, without loss of consciousness or pain during these and favourable response to antiepileptic drugs [13]. In the families with PKD, a spontaneous improvement or remission was found at the beginning of adulthood. Conversely, in the family with PED, the attacks persisted even at advanced ages. The effective response to levodopa in the family with PED and the favourable outcome with ketogenic diet in the sporadic case with PED leads us to think that we could be in front of two distinct genetic forms, GTP-cyclohydrolase, and GLUT-1 deficiencies, respectively [5, 14, 15]. In the cases diagnosed as PNKD, the patients were recommended to avoid consumption of alcohol and coffee [1], but no treatment was tested given the low frequency and short duration (1/month, 5 min) of the episodes.

On the other hand, the present study describes 9 cases of PxD associated with a specific cause. These results suggest that secondary forms may be responsible for more cases of PxD (40%) than reported previously (22%) [7]. The secondary forms of PxD differ from primary forms in the variable duration of symptoms in PNKD (2-60 min), the wide range of ages of onset (27-81 years), the overlap of kinesigenic and nonkinesigenic symptoms in one case [S2], and the frequent existence of neurological deficits in the interictal periods [S1, 3, 6, 8]. The response to treatment is difficult to interpret, especially for PNKD. In the patients with multiple sclerosis (MS) and cerebral haemorrhage was observed an improvement with anticonvulsants and benzodiazepines, respectively, outcomes consistent with other cases published [16–18]. However, in two cases with encephalitis (one in a patient with HIV stage C3), neither benzodiazepines nor anticonvulsants were beneficial, not confirming the effective response to benzodiazepines observed in some patients with HIV from the literature [19]. In the case with PKD/PNKD of vascular aetiology, levodopa was useful; and in that one with PED due to peripheral trauma, trihexyphenidyl resulted effective, supporting descriptions of previous isolated cases [20]. Notwithstanding, the identification of the underlying cause is the most important thing, because the proper treatment aimed to the cause can reverse symptoms, as it was found in patients with metabolic disorders and iatrogenic of this series [7].

The most frequent causes of PxD reported until the date are in line with the presented study (MS, vascular lesions, trauma, metabolic disorders, and central nervous system infections [CNS]) [21].

The patient with MS underwent the typical paroxysmal painful hemidystonia preceded by aura described in the onset or course of MS, but in our case there were no triggers such as hyperventilation or tactile stimulation [22–24]. PxD in MS are associated with lesions in any part of the CNS and are attributed to alterations of ephaptic transmission [17, 25]. PxD symptoms in the vascular aetiology correspond to the infarction area and can be reversed with interventions that increase the cerebral perfusion in case of ischemia [7]. It is also essential to recognize the PxD in cases with HIV, since they may be the result of opportunistic infections, as it happens in the patient presented [26]. Nevertheless, 6 cases of HIV infection in advanced stages without coexisting opportunistic infections had been reported in the literature, one with a pathological study which revealed encephalitis due to severe HIV as well as loss of positive neurons to calbindin in the basal ganglia [19, 27]. Finally, especially striking is the cases of peripheral trauma and iatrogenic [28–30]. While the mechanism underlying the peripheral trauma-induced PxD is unknown, it has been suggested that peripheral lesions can lead to aberrant cortical and spinal reorganization, which would explicate the observed motor dysfunction—PxD included—after the peripheral injury. However, for some authors, the relationship between peripheral injury and PxD remains highly controversial [31, 32]. In the case reported in our series, a psychogenic origin was excluded due to the absence of atypical clinical features or response to suggestion in the witnessed episodes provoked by exercise. In the iatrogenic cases, the emergence of PxD coincided with the start of escitalopram or fluoxetine and the PxD disappeared immediately after withdrawal of selective serotonin reuptake inhibitors (SSRIs); i.e., it meets the temporal pattern of movement disorders induced by drugs. SSRIs may play an indirect antidopaminergic effect, which could explain the development of PxD [33, 34].

## 5. Conclusion

Paroxysmal dyskinesias constitute an entity that must be considered in the differential diagnosis of movement disorders. Thus, despite its unusual appearance, it is likely that these disorders are underdiagnosed and, consequently, the patients suffer a delay in diagnosis and treatment, as well as undergoing various unnecessary additional tests.

## Figures and Tables

**Table 1:** Summary of the results of cases sample identified as primary paroxysmal dyskinesias.

Case number	Sex	Age at onset (years)	Current age (years)	Predominant movement	Duration	Frequency	Aura	Location	Precipitants or exacerbating factors	Treatment	Evolution
F1.I.1	M	12	71	D	15-20 secs	1-2/month	Yes	U: Left hand	Sudden movement, startle	–	Remission at 25 years

F1.II.1	M	9	48	CD	30-60 sec	2/week	Yes	U: Left arm and leg	Sudden movement	–	Remission at 25 years

F1.II.7	F	8	24	CD, anartria	1-2 min	20-30/day	Yes	A: Left>right arm and leg; face	Sudden movement, stress, fatigue, startle	TPM, CBZ, LTG	No attacks with TPM (weight loss) and LTG; CBZ: 2/week

F1.III.1	F	5	6	CD	30-60 sec	3-4/day	No	A: Hemi-corporal	Sudden movement	–	No changes

F2.III.2	M	8	32	D	30-60 sec	1/day	No	U: Right hand	Sudden movement	–	Remission at 20 years

F2.III.3	F	7	30	D	1-2 min	2/day	Yes	A: Hands	Sudden movement, stress	LTG	Removing LTG without attacks

F3.I.1	M	25	75	D	10 min	4/week	No	B: Hands	Exercise	–	No changes

F3.II.2	F	28	50	D	15-60 min	1/day	No	A o B: Feet > hands	Exercise, stress	ACTZ,CZP, PGB, L-dopa	Improvement with L-dopa

F3.III.2	F	20	23	D	15 min	2/day	No	B: Hands	Exercise	–	No changes

E1	F	23	27	D	30-60 sec	5/day	Yes	U: Right hand	Sudden movement	TPM	Improvement with TPM

E2	M	16	18	D	5 min	1-2/month	Yes	A: Hands	Rest	–	No changes

E3	M	33	34	D	5 min	1/month	No	U: Left hand	Rest, alcohol	–	No changes

E4	F	7	14	D	10 min	1-2/day	No	U: Left arm and leg	Exercise; fatigue, hunger	Ketogenic diet	Improvement with diet

^a^At the time with the larger number of episodes; F: familial; E: sporadic; M: male; F: female; D: dystonia; CD: mixed chorea and dystonia; A: alternant (sometimes affects one side and others to the other one); U: unilateral; B: affects both sides simultaneously; TPM: topiramate; CBZ: carbamazepine; LTG; lamotrigine; ACTZ: acetazolamide; CZP: clonazepam; PGB: pregabalin.

**Table 2:** Summary of the results of cases sample identified as secondary paroxysmal dyskinesias.

Case number/ Cause	Sex	Age at onset (years)	PxD	Predominant movement	Duration	Frequency	Aura	Location	Precipitants or exacerbating factors	Treatment	Evolution
S1/ MS	F	32	PNKD	D	2-5 min	3/day	Yes	U: Right hand	Rest	OXC	Remission in 1 month

S2/ CVD	F	60	PKD/PNKD	D	2-3 min	4/week -> 2-3/day	No	G or U: Left hand or foot	Rest, sudden movement	L-dopa	Improvement with L-dopa

S3/ HIV, encephalitis	M	29	PNKD	CD	<5 min	10-20/day	No	U: Left arm and leg, face, trunk	Rest	^(1)^CZP, DPH, VPA, CBZ, LEV iv	No improvement. Death after 2 months by pneumonia

S4/ fluoxe-tine	F	34	PNKD	D	3-5 min	1-5/day	Yes	U: Left arm and leg	Rest, stress	Remove SSRIs, BZD	Remission after withdrawal

S5/ escita-lopram	M	27	PNKD	D	15 min	3/day	No	U: Left hand	Hand rest, walking	Remove SSRIs, GBP	Remission alter withdrawal

S6/ fungal encephalitis	M	81	PNKD	CD	30-60 min	5-10/ day	No	U: Right arm and leg	Rest	^(1)^CZP, DPH, LEV iv	No improvement. Death after 1 day

S7/ hipoPTH	F	68	PKD	D	< 1 min	1/week	No	A: Hands or feet	Sudden movement	Calcium	Improvement with calcium

S8/ haemorrhage	F	77	PNKD	C	<5 min	10-20/day	No	U: Left arm and leg	Rest	CZP iv	Remission in 1 week

S9/ peripheral trauma	F	51	PED	CD	15-30 min	2-4/day	No	U: Right foot	Exercise	TBZ, CZP, trihexyphenidyl	Improvement with trihexyphenidyl

^a^At the time with the larger number of episodes; S: secondary; MS: multiple sclerosis; CVD: cerebrovascular disease; PTH: parathyroidism; M: male; F: female; D: dystonia; CD: mixed chorea and dystonia; A: alternant (sometimes affects one side and others to the another one); U: unilateral; G: generalized; OXC: oxcarbazepine; CZP: clonazepam; DPH: difenilhidantoina; VPA: valproic acid; CBZ: carbamazepine; LEV: levetiracetam; IV: intravenous; SSRIs: selective serotonin reuptake inhibitors; BZD: benzodiazepines; GBP: gabapentin; TBZ: tetrabenazine.

^(1)^Besides the etiological treatment: antiretroviral agents (lopinavir/ritonavir; emtricitabine/tenofovir), antitoxoplasma therapy and steroids in S3, and antifungals in S6.

## Data Availability

The data is available on request from the author.
